# Virus and Host Determinants of West Nile Virus Pathogenesis

**DOI:** 10.1371/journal.ppat.1000452

**Published:** 2009-06-26

**Authors:** Michael S. Diamond

**Affiliations:** Departments of Medicine, Molecular Microbiology, and Pathology & Immunology, Washington University School of Medicine, St Louis, Missouri, United States of America; University of California San Francisco, United States of America

## Changes to the West Nile Virus Genome Have Increased Its Virulence in Birds and Allowed Rapid Spread

West Nile virus (WNV) is a small, enveloped, mosquito-transmitted, positive-stranded RNA virus of the Flaviviridae family. This virus is related to other arthropod-borne viruses that cause human disease globally, including dengue, yellow fever, and Japanese encephalitis viruses. WNV cycles in nature primarily between *Culex* mosquitoes and birds, but also infects human, horses, and other vertebrates. Over the latter half of the 20th century, outbreaks of WNV infection have been reported in Europe, Asia, and Australia. In 1999, WNV was introduced into the Western Hemisphere in New York City.

Early during the WNV epidemic in the United States, it became apparent that certain species of birds, including crows, blue jays, and ravens, were vulnerable to lethal infection. This phenotype was not described in prior outbreaks in other parts of the world. Genome sequencing combined with reverse genetic approaches has provided some insight as to why WNV became virulent for some avian species. Although the exact molecular mechanism remains uncertain, a single amino acid change in the NS3 helicase in North American WNV isolates gene associates with pathogenesis in crows [1].

After its entry into North America, there was an initial period of sequence conservation among strains. However, by 2002, two sequence subtypes were detected, one of which differed in the envelope (E) protein at amino acid 159 (WNV 2002). WNV 2002 has emerged as the dominant WNV genotype in North America. Experiments in mosquitoes have begun to explain why this strain displaced WNV 1999 and promoted the rapid spread across the continent. The change at residue 159 allows greater viral replication in the mosquito at higher temperatures, which translates into higher transmission of the virus to birds, the natural vertebrate host of WNV [2],[3]. Thus, a single amino acid change in WNV has led to rapid geographic expansion and increased intensity of transmission.

## Paradoxes of the Humoral Immune Response against WNV

Humoral immunity limits WNV dissemination, as animals lacking antibodies develop high-grade viremia, early dissemination into the brain, and uniform mortality [4]. The envelope E glycoprotein is the major surface structural protein on the virion and the principal antigen that elicits neutralizing antibodies; as such, it is a primary target for vaccine development. New studies suggest and explain why the level of neutralizing antibodies in serum may not always correlate with protection against WNV.

WNV virions incorporate 180 E proteins on their surface. The E proteins of newly synthesized immature WNV are organized into trimeric spikes composed of pre-membrane (prM) and E protein heterodimers. During egress, immature virions undergo a maturation step in which a furin-like protease cleaves prM, resulting in a reorganization of E proteins into a distinct homodimeric array [5]. As complete maturation is not required for infectivity, WNV virions can be heterogeneous with respect to the level of prM on their surface. In theory, mature, partially mature, and mostly immature virions may be differentially recognized and neutralized by antibodies. Recent experiments establish that virion maturation impacts the sensitivity of WNV to antibody neutralization [6]. Antibodies that target the virus fusion peptide, which comprise a major component of the human antibody response following WNV infection or vaccination, do not neutralize the fully mature form of the virion.

Interestingly, some poorly neutralizing human antibodies against E may still be protective in vivo (M. Vogt, M. Throsby, and M. Diamond, unpublished data), presumably due to interactions with complement and/or Fc-γ receptors. Furthermore, NS1, a non-structural glycoprotein that is absent from the virion but expressed on the surface of infected cells, elicits non-neutralizing protective antibodies that inhibit infection through both Fc-γ receptor–dependent and -independent mechanisms [7]. Although both neutralizing and non-neutralizing antibodies contribute to humoral protection against WNV infection and possibly other flaviviruses, current vaccine programs judge the efficacy of the humoral response primarily on the magnitude of neutralizing antibodies measured in serum. The studies outlined above suggest that improved correlations with protection in vivo may be achieved if the maturation state of the virion, the functional profile of anti-NS1 antibodies, and effector mechanisms of antibodies are considered.

## A Protective Cellular Immune Response against WNV in the Brain

For many encephalitic viruses, the cellular immune response in the central nervous system (CNS) can be pathologic, as injury to neurons or glial cells causes irreversible loss of function and death [8]. However, cellular immunity is absolutely required for clearance of infection by virulent North American WNV isolates. Mice deficient in CD4^+^ or CD8^+^ T cells develop persistent WNV infection in the brain [9],[10], and humans with hematological malignancies and impaired T cell function are at increased risk of neuroinvasive WNV infection and poor outcome [11]. WNV-specific CD8^+^ T cells secrete inflammatory cytokines and lyse virus-infected neurons through perforin- and Fas ligand–dependent mechanisms, whereas CD4^+^ T cells sustain WNV-specific CD8^+^ T cell responses in the CNS that enable viral clearance [10]. This lytic and potentially destructive activity of WNV-specific CD8^+^ T cells in the brain is paradoxically protective because of the urgent need to limit replication of a highly cytopathic virus in neurons. Consistent with this, if mice are infected with a less pathogenic isolate of WNV, the CD8^+^ T cell response can be detrimental and cause excessive immunopathogenesis and injury [12].

## West Nile Virus and IFN: Regulation and Counter-Regulation

The interferon (IFN) response is an essential host defense program that limits infection of many families of RNA and DNA viruses. IFN-α and -β are produced during the earliest stages of WNV infection after host cell recognition of viral RNA. Mammalian cells detect WNV viruses and induce IFN by recognizing single (ssRNA) or double-stranded (dsRNA) viral RNA through the endosomal Toll-like receptors 3 and 7 (TLR3 and TLR7), and the cytoplasmic proteins retinoic acid-inducible gene I (RIG-I) and melanoma-differentiation-associated gene 5 (MDA5). Mice with genetic defects in any of these individual RNA sensors, the receptor for IFN-α and -β, or constituents of its signaling cascade (e.g., STAT1) show markedly enhanced viral burden in tissues, which leads to rapid lethality.

**Figure 1:**
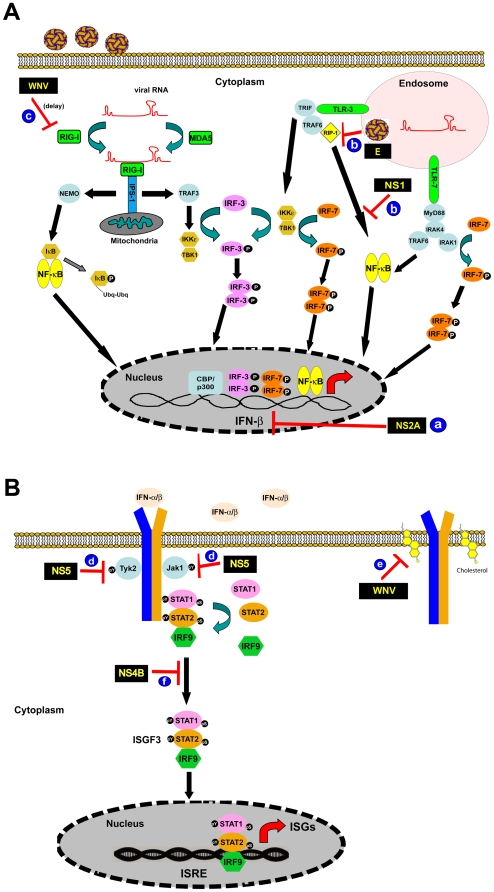
IFN signaling and mechanisms of evasion by WNV. (A) Infection by WNV produces dsRNA intermediates within the cytoplasm that display motifs recognized by the RIG-I and MDA5 helicases. Binding of viral RNA promotes an interaction with IPS-1 that results in recruitment of signaling proteins (e.g., NEMO and TRAF3) that lead to activation of IRF-3 and NF-κB. These factors translocate to the nucleus and bind to the promoter region of the IFN-β gene, leading to transcription and translation. In some cells, TLR3 and TLR7 in endosomes recognize dsRNA and ssRNA motifs, leading to recruitment of cytoplasmic adaptor molecules (MyD88 and TRIF), which initiate signaling cascades that activate IRF-3, IRF-7, and NF-κB, resulting in IFN-β gene transcription. Mechanisms of evasion by WNV include the following: (a) reduction in IFN-β gene transcription by the viral NS2A protein; (b) impairment of RIP-1 signaling by high mannose carbohydrates on the structural E protein and attenuation of TLR3 signaling by NS1; and (c) a delay in recognition of WNV RNA by RIG-I. (B) Secretion of IFN by WNV-infected cells results in autocrine and paracrine signaling through the heterodimeric receptor for IFN-α and β (IFNAR). Binding by IFN results in activation and tyrosine phosphorylation of JAK family members (JAK1 and Tyk2) and the cytoplasmic tail of the IFN-αβR. This promotes recruitment of the STAT1 and STAT2, which themselves become phosphorylated by the JAKs. Phosphorylated STAT1 and STAT2 proteins heterodimerize, associate with IRF-9, and translocate to the nucleus, where they bind IFN-stimulated response element (ISRE) sequences to induce expression of hundreds of ISGs. Mechanisms of evasion by WNV include (d) blockade of phosphorylation of Tyk2 and JAK1 by NS5; (e) down-regulation of the IFNAR through virus-induced redistribution of cellular cholesterol; and (f) attenuation of STAT signaling by NS4B.

Although IFN restricts infection, WNV has evolved countermeasures to limit its efficacy. Indeed, WNV is resistant to the antiviral effects of IFN in cell culture once infection is established. This may explain the relatively narrow therapeutic window for IFN administration that has been observed in animal models or humans infected with WNV [13]. WNV attenuates IFN function at multiple steps of the induction (Figure 1A) and signaling (Figure 1B) cascade. (a) The WNV NS2A protein inhibits IFN-β gene transcription [14]. (b) WNV E and NS1 proteins independently inhibit IFN-β transcription in response to dsRNA [15],[16]. (c) Pathogenic WNV strains evade IRF-3-dependent signals without actively antagonizing the host defense signaling pathways [17]. This provides a kinetic advantage to elude the IFN response at early times after infection. WNV also targets the JAK-STAT proteins, essential elements of the IFN signaling pathway, to prevent the induction of antiviral genes. Thus, even when type I IFN is produced, it may not achieve its inhibitory effect. (d) WNV interferes with phosphorylation of JAK1 and Tyk2 [18]. (e) WNV infection actively promotes re-localization of cholesterol, which diminishes the formation of cholesterol-rich lipid rafts in the plasma membrane and attenuates the IFN signaling [19]. (f) The NS4B of WNV partially blocks STAT1 activation and IFN-stimulated gene (ISG) induction [20].

## The Genetic Basis of Risk of Human WNV Infection Is Beginning to Be Understood

Most (∼80%) human WNV cases develop without significant symptoms. Among clinically apparent cases, many experience a self-limited, although at times debilitating, febrile illness. A subset of WNV cases progress to paralysis, meningitis, and/or encephalitis.

Two human genes have been identified as susceptibility loci for WNV infection. (1) ***CCR5***. Experiments in animals had established that chemokines direct leukocytes to the brain to clear WNV from infected neurons. In mice, a genetic deficiency of the chemokine receptor CCR5 was associated with depressed leukocyte trafficking, increased viral burden, and enhanced mortality [21]. Interestingly, analogous genetic deficiencies (e.g., *CCR5Δ32*, a deletion in the *CCR5* gene) are associated with WNV-induced disease in humans [22]. Although individuals that are homozygous for the *CCR5Δ32* allele represent about ∼1% of the general United States population, 4%–8% of individuals with laboratory-confirmed symptomatic WNV infection were homozygous for the mutant allele. Thus, CCR5 functions as an essential host factor to resist neuroinvasive WNV infection, which may have implications for the use of CCR5 antagonists in HIV therapy. (2) ***OAS***. In certain mouse strains, susceptibility to flaviviruses, including WNV, maps to a truncated isoform of the 2′ 5′ oligoadenylate sythetase (*OAS1b*) gene, a member of an IFN-regulated gene family involved in degradation of viral RNA. A recent study suggests that a hypomorphic allele of the human ortholog *OAS1* is associated with both symptomatic and asymptomatic WNV infection [23]. This data suggests that in humans, *OAS1* is a genetic risk factor for initial WNV infection, although not for disease severity.

## Conclusions

Pathogenesis and host immune response studies with model viruses (e.g., lymphocytic choriomeningitis, polio, herpes simplex, and mouse hepatitis viruses) created an impression that the virus–host interface is remarkably conserved, and the paradigms generated for one pathogen are readily transferable to another. However, the complexity of individual viruses with respect to enzootic cycles, the host–pathogen–vector interface, cellular tropism, structural diversity, ability to induce injury, and immune system recognition and evasion has established that the exception is often the rule: the host responds differently to control individual viruses. Studies with WNV are continuing to educate us as to the extent of this complexity. Hopefully, these lessons will translate into novel therapeutics and vaccines against WNV and possibly, closely related pathogenic flaviviruses.

## References

[1] Brault AC, Huang CY, Langevin SA, Kinney RM, Bowen RA (2007). A single positively selected West Nile viral mutation confers increased virogenesis in American crows.. Nat Genet.

[2] Moudy RM, Meola MA, Morin LL, Ebel GD, Kramer LD (2007). A newly emergent genotype of West Nile virus is transmitted earlier and more efficiently by Culex mosquitoes.. Am J Trop Med Hyg.

[3] Kilpatrick AM, Meola MA, Moudy RM, Kramer LD (2008). Temperature, viral genetics, and the transmission of West Nile virus by Culex pipiens mosquitoes.. PLoS Pathog.

[4] Diamond MS, Pierson TC, Fremont DH (2008). The structural immunology of antibody protection against West Nile virus.. Immunol Rev.

[5] Mukhopadhyay S, Kim BS, Chipman PR, Rossmann MG, Kuhn RJ (2003). Structure of West Nile virus.. Science.

[6] Nelson S, Jost CA, Xu Q, Ess J, Martin JE (2008). Maturation of West Nile virus modulates sensitivity to antibody-mediated neutralization.. PLoS Pathog.

[7] Chung KM, Nybakken GE, Thompson BS, Engle MJ, Marri A (2006). Antibodies against West Nile virus non-structural (NS)-1 protein prevent lethal infection through Fc gamma receptor-dependent and independent mechanisms.. J Virol.

[8] McGavern DB, Homann D, Oldstone MB (2002). T cells in the central nervous system: the delicate balance between viral clearance and disease.. J Infect Dis.

[9] Shrestha B, Diamond MS (2004). The role of CD8+ T cells in the control of West Nile virus infection.. J Virol.

[10] Sitati E, Diamond MS (2006). CD4+ T Cell responses are required for clearance of West Nile Virus from the central nervous system.. J Virol.

[11] Murray K, Baraniuk S, Resnick M, Arafat R, Kilborn C (2006). Risk factors for encephalitis and death from West Nile virus infection.. Epidemiol Infect.

[12] Wang Y, Lobigs M, Lee E, Koskinen A, Mullbacher A (2006). CD8(+) T cell-mediated immune responses in West Nile virus (Sarafend strain) encephalitis are independent of gamma interferon.. J Gen Virol.

[13] Chan-Tack KM, Forrest G (2005). Failure of interferon alpha-2b in a patient with West Nile virus meningoencephalitis and acute flaccid paralysis.. Scand J Infect Dis.

[14] Liu WJ, Wang XJ, Clark DC, Lobigs M, Hall RA (2006). A single amino acid substitution in the West Nile virus nonstructural protein NS2A disables its ability to inhibit alpha/beta interferon induction and attenuates virus virulence in mice.. J Virol.

[15] Scholle F, Mason PW (2005). West Nile virus replication interferes with both poly(I:C)-induced interferon gene transcription and response to interferon treatment.. Virology.

[16] Wilson JR, de Sessions PF, Leon MA, Scholle F (2008). West Nile virus nonstructural protein 1 inhibits TLR3 signal transduction.. J Virol.

[17] Fredericksen BL, Gale M (2006). West Nile virus evades activation of interferon regulatory factor 3 through RIG-I-dependent and -independent pathways without antagonizing host defense signaling.. J Virol.

[18] Guo JT, Hayashi J, Seeger C (2005). West Nile virus inhibits the signal transduction pathway of alpha interferon.. J Virol.

[19] Mackenzie JM, Khromykh AA, Parton RG (2007). Cholesterol manipulation by West Nile virus perturbs the cellular immune response.. Cell Host Microbe.

[20] Munoz-Jordan JL, Laurent-Rolle M, Ashour J, Martinez-Sobrido L, Ashok M (2005). Inhibition of alpha/beta interferon signaling by the NS4B protein of flaviviruses.. J Virol.

[21] Glass WG, Lim JK, Cholera R, Pletnev AG, Gao JL (2005). Chemokine receptor CCR5 promotes leukocyte trafficking to the brain and survival in West Nile virus infection.. J Exp Med.

[22] Glass WG, McDermott DH, Lim JK, Lekhong S, Yu SF (2006). CCR5 deficiency increases risk of symptomatic West Nile virus infection.. J Exp Med.

[23] Lim JK, Lisco A, McDermott DH, Huynh L, Ward JM (2009). Genetic variation in OAS1 is a risk factor for initial infection with West Nile virus in man.. PLoS Pathog.

